# Who are the most affected by *Bothrops* snakebite envenoming in Brazil? A Clinical-epidemiological profile study among the regions of the country

**DOI:** 10.1371/journal.pntd.0011708

**Published:** 2023-10-19

**Authors:** Weslley Ruan Guimarães Borges da Silva, Lucas de Siqueira Santos, Derick Lira, Karla Patrícia de Oliveira Luna, Sayonara Maria Lia Fook, Rômulo Romeu Nóbrega Alves

**Affiliations:** 1 Department of Biology, Center of Biological and Health Sciences, Paraíba State University, Campina Grande, Paraíba, Brazil; 2 Graduate Program in Bioinformatics, Department of Biophysics and Pharmacology, Federal University of Rio Grande do Norte, Natal, Rio Grande do Norte, Brazil; 3 Graduate Program in Geodetic Sciences and Geoinformation Technologies, Department of Cartographic Engineering, Federal University of Pernambuco, Recife, Pernambuco, Brazil; 4 Graduate Program in Ecology and Conservation, Department of Biology, Paraíba State University, Campina Grande, Paraíba, Brazil; 5 Graduate Program in Science Teaching and Mathematics Education, Department of Biology, Paraíba State University, Campina Grande, Paraíba, Brazil; 6 Graduate Program in Cellular and Molecular Biology, Department of Molecular Biology, Federal University of Paraiba, João Pessoa, Paraíba, Brazil; 7 Graduate Program in Public Health, Department of Pharmacy, State University of Paraíba, Campina Grande, Paraíba, Brazil; 8 Graduate Program in Ethnobiology and Nature Conservation, Federal Rural University of Pernambuco, Recife, Pernambuco, Brazil; Monash University, AUSTRALIA

## Abstract

Snakebite envenoming represents an important Neglected Tropical Disease (NTD) that mainly affects tropical and subtropical developing countries according to the World Health Organization (WHO). As a priority issue in the tropics, it is estimated that accidental encounter between snakes and humans is the leading cause of morbidity and mortality among all NTDs in the world. In Brazil, an extremely diverse country with continental dimensions, snakebite envenoming is the second leading cause of reported human envenoming. Treating the disease has been an unprecedented challenge for Brazilian Health Systems for decades. Despite access to Antivenom therapy and distributing it free of charge across the country, Brazil faces numerous issues regarding the notification process and accurate treatment targeting for at-risk populations. Thus, this study aimed to identify the temporal epidemiological dynamics of accidents caused by *Bothrops* snakes in Brazil, the country’s major group of venomous snakes, based on secondary information from the online database provided by The Brazilian Notifiable Diseases Information System (SINAN). For this purpose, reported *Bothrops* snakebites between 2012 and 2021 were counted, then the data were analyzed. We looked at the frequency, occurrence, mortality rates, case fatality rate (CFR), age and gender distribution, and the time lapse between the incident and the initiation of Antivenom therapy. The data were also organized considering regional variations of the country. Throughout the studied period, a total of 202,604 cases of envenoming caused by *Bothrops* spp. were notified, resulting in 766 fatalities. These accidents were found to occur in variable proportions across different regions in Brazil, with notable concentrations observed in the North, Northeast, and Southeast regions. The epidemiological profile of patients varied greatly between the regions, revealing that snake envenoming is much more a social, economic, and ecological problem than a medical one. In conclusion, our study provides an overview of the clinical and epidemiological profile of envenoming by *Bothrops* snakes in Brazil. Notably, this is the first study to present such information in a country as vast and diverse as Brazil, encompassing a comparative analysis of its regions using SINAN data, that proves to be a very useful national tool to improve the control and management of envenoming.

## Author summary

Snakebite envenoming it is a health challenge worldwide. In Brazil, the largest country in Latin America, *Bothrops* snakebites represents the main cause of envenoming by venomous snakes. Every year, at least 20,000 accidents with *Bothrops* snakes are notified by The Brazilian Information System for Notifiable Diseases (SINAN). However, it is well known that these numbers do not represent reality in many cases, since a significant portion of envenoming patients continue to dispense specialized medical care or are unable to arrive in time at the reference medical centers. Therefore, in our comprehensive study with secondary data made available online by SINAN, we decided to understand the temporal dynamics of *Bothrops* snakebites over a ten-year period across the five regions of Brazil. According to our results, which evaluated variables such as occurrence, incidence, mortality and lethality, envenoming by *Bothrops* snakes remains an important neglected public health problem in our country. Currently, the high rates of occurrence and incidence in certain regions are due to socioeconomic problems found in a vast and developing country such as Brazil. Despite this, lethality and mortality rates remain low at all regions of the country, a fact that can be associated with the effectiveness of the Antivenom therapy treatment provided free of charge by The Brazilian National Health System (SUS).

## 1. Introduction

Snakebite envenoming represents a neglected public health disease that mainly affects tropical and subtropical developing countries [1–6]. Worldwide, it is estimated that the accidental encounter between snakes and humans is the main cause of morbidity and mortality among all Neglected Tropical Diseases (NTDs) listed by World Health Organization such as priority diseases in the tropics [7–9].

Annually, 5.4 million snakebites are reported in the world, that results in 1.8 to 2.7 million envenoming and 81,000–138,000 deaths [9–11]. Despite this, the true incidence of global snakebites remains unknown due to underreporting [8,12]. Among those affected are mainly agricultural workers and children, who lives in poorly built houses, normally, remote areas from access to education and health care centers [3,13]. As result, snakebite envenoming is treated as an occupational disease with a social and economic nature [10].

Evaluation of the incidence and mortality, as well as the identification of regions at risk is necessary to design mapping and surveillance measures [14]. These measures must assist in developing of new strategies for diagnosis, monitoring and treatment, aiming at the prevention and reduction of these accidents, as well as the promotion of therapeutic actions for the control of the disease [5,14–17], which include offering Antivenom to neutralize the effects of toxins in the blood of envenoming patients [18–21].

Methods capable of mapping epidemiological scenarios have existed for decades, but they have not been sufficient to identify the entire situation related to snakebite envenoming [22,23]. In this context, statistical and mathematical models capable of estimating and predicting epidemiological patterns have been developed [24]. However, many of these data are grouped on country-level scales, which on several occasions makes it difficult to carry out more accurate analyses for the envenoming in certain countries, such as Brazil [5,13,17,25].

Brazil is considered a model country for epidemiological surveys on snakebite envenoming, according to its high zoological, ecological [13,26], climatic [27,28] and socioeconomic diversity [29,30]. Only between 2011 and 2020, 1.9 million accidents with snakes were recorded in this country, of which at least 1.7 million were caused by unidentified snakes, 17,000 accidents by non-venomous snakes and 235,872 by venomous snakes such as *Bothrops* spp., the major group of venomous snakes in this country [31–33].

The Neotropical genus *Bothrops* is an assemblage of about 48 species distributed from Mexico to Argentina [33]. These animals are found in a variety of habitats, from tropical rainforests, lowland or mountainous areas, grasslands, and dry habitats [34–36]. Namely, 30 species of the genus can be found in Brazil [33]. Due to its size and biodiversity, the Amazon region is home for many of them [37,38], however only *Bothrops atrox* is responsible for about 80 to 90% of the accidents in this region [39].

In certain areas of Northeast of the Brazil, *B*. *erythromelas* has been identified as the main agent of snakebite envenoming [27,40–42]. In the South and Southeast, ecological and toxicological research with *B*. *jararaca* is performed due to its medical significance in snakebite envenoming to these regions [43,44]. For the Brazilian Cerrado regions, Midwest region of Brazil, *B*. *moojeni* is commonly found, and its accidental interaction with humans can lead to envenoming cases [45].

Despite the acknowledged significance of *Bothrops* snakebites, there remains a pressing need for heightened attention towards this issue. One key exacerbating factor, with a global impact, is the limited focus and support from funding organizations, public health authorities, pharmaceutical industry stakeholders, and health advocacy groups. This lack of attention hinders the development of effective interventions, leading to increased impracticality and difficulty in addressing the problem more assertively [10].

To monitor, analyse and improve the understanding of the occurrence of human envenoming in Brazil, the Health Information Systems (SIS) are used in the collection and storage of epidemiological data [46]. Introduced in 1998 [47], The Brazilian Notifiable Diseases Information System (SINAN) plays a pivotal role in monitoring snakebite envenoming. Since its inception, SINAN has become the crucial for at least two thousand health care centers dedicated to envenoming, to conduct new research studies, developing strategies and prevention measures [48,49].

Thus, identifying the temporal epidemiological dynamics of *Bothrops* snakebites in Brazil can be done based on the online database provided by the SINAN, and will help in making decisions to improve management and treatment of the disease. Based on this, three important questions can be raised and should serve as the basis for this study: (1) What are the populations that lack a political voice and suffer most from *Bothrops* snakebites in Brazil? (2) What has been the temporal therapeutic itinerary taken by patients and how has this influenced the management and treatment of the envenoming? (3) How can these data help the Ministry of Health’s National Zoonosis Surveillance and Health programs?

## 2. Methods

### 2.1 Ethical aspects

The SINAN and IBGE databases, which are in the public domain, do not contain identifiable information regarding individuals. In 2016, a new resolution published by the National Health Council (CNS-BR) revoked the need to seek approval from any Research Ethics Committee for studies using publicly available secondary data that do not provide individually identifiable information (http://conselho.saude.gov.br/resolucoes/2016/reso510.pdf), since publicly accessible information can be used in the context of research and in the transmission of knowledge without restricting the access of researchers and citizens in general, not being subject to limitations related to privacy, security or access control. Thus, supported by the terms established by Resolution of the National Health Council No. 510, of April 7, 2016, it was not necessary to request approval of the research by an ethics committee.

### 2.2 Study area

Brazil is the largest country in Latin America in terms of land area (8,515,767 km^2^) [50] and is home to a population of 190,755,799 individuals, as per the most recent demographic census, conducted in 2010 by the Brazilian Institute of Geography and Statistics [51]. The country is divided into five major administrative regions and comprises 26 states, along with the Federal District. Most of the Brazilian population is concentrated in urban areas, with only 15.6% residing in rural regions. However, regional disparities exist, as approximately 27% of the population in the North and Northeast regions live in rural areas, compared to just 7.1% in the Southeast. Additionally, around 3% of the total population comprises agricultural workers [51] Discrepancies in literacy rates are also evident, ranging from 95.9% in the South to 83.8% in the Northeast Region, with an average of 92.0% for the country [52]. Similarly, significant variations in Gross Domestic Product (GDP) per capita are observed, with figures ranging from R$34,790 in the Southeast to R$12,955 in the Northeast [53].

### 2.3 Data collection, population and study design

Information about *Bothrops* envenoming in Brazil between 2012 and 2021 was collected from The Brazilian Notifiable Diseases Information System (SINAN) of the Brazilian Ministry of Health. In this system, the identification of snake genus relied on information provided by the victim or their companion, as well as on observations of signs and clinical symptoms noted by health officials, that use, routinely, the guidelines for identification purposes found in Venomous Animal Accident Treatment Manual, provided by The Brazilian National Health Foundation [54] and the guidelines found in Health Surveillance Guide of the Brazilian Ministry of Health [55]. In Brazil the envenoming data is collected in toxicological centers from Notification Forms (http://portalsinan.saude.gov.br/Sinan). To our analyzes we collected (a) number of cases per year according to the state of notification; (b) ethnicity, gender and age range of admitted patients; (c) time elapsed between the accident and health care center; (d) presence of local manifestations and complications; and (e) presence of systemic manifestations and complications.

### 2.4 Statistical data analysis

All data collected were transferred to Excel and analyzed in R software. Trend curves and R^2^ correlation indices were calculated, and the significance level was equal to 0.05. The incidence of cases was identified based on the number of accidents reported during the study period divided by the number of the population of each state/region x 100,000 inhabitants [56]. To verify differences between snakebite envenoming in populations defined by male and female gender, we used the chi-square test (x^2^). Therefore, we considered the total number of snake envenoming per region over the years as a response variable and gender as an explanatory variable. Allied to this, to verify differences in the occurrence of envenoming between years by region and age groups, a non-parametric Kruskal-Wallis (H) test was used followed by a post-hoc Dunn test. In the first test, we evaluated the total number of snake envenoming between 2012 and 2021 by region, and from the second test, we compared envenoming by age group using the following sample sizes: under 1 year old, from 1–4, 5–9, 10–14, 15–19, 20–39, 40–59, 60–64, 65–69, 70–79 and over 80 years old.

### 2.5 Access to mortality and lethality rates

Mortality rates (1) per 100,000 inhabitants and lethality (2) (%) were calculated according to the equations:

$$(1)\;Mortality\;=\;\frac{(number\;of\;deaths)\cdot 100,000}{population}$$

$$(2)\;Lethality=\;\frac{(number\;of\;deaths)\cdot \;100}{\left(number\;of\;cases\right)}$$

### 2.6 Graphic resources and maps

The maps that show the spatial distribution of occurrences and incidence rates of cases of envenoming were made using the open-source software QGIS, version 3.16.10. The choice of intervals represented on the maps was made by rounding off the intervals generated by Jenks’ classification by natural breaks [57]. The method aims at minimizing variance within groups of similar values and maximizing differences between different groups. In this way, the method used becomes ideal for representing data that have an irregular distribution between them. Data regarding the spatial distribution of reference centers for handling accidents caused by *Bothrops* spp. were obtained from Brasil [58], and the addresses were geocoded (i.e., transformed into geographical coordinates) using the Vilimpoc et al. code [59], which links a Google Sheets macro to the Google Maps geocoding API to automatically extract addresses.

## 3. Results

### 3.1 Epidemiological profile of accidents with *Bothrops* in Brazil

**Fig 1:**
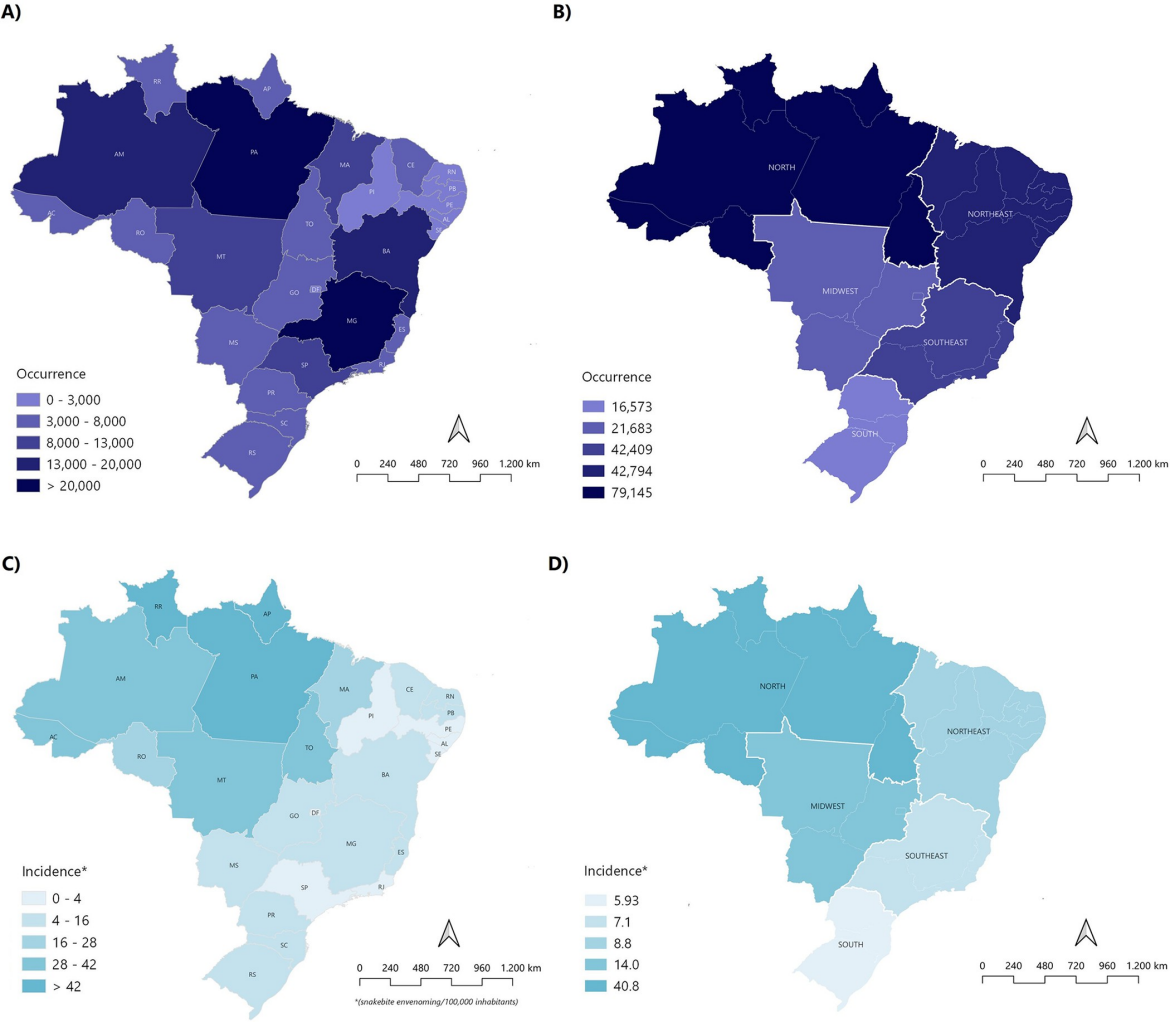
Occurrence and incidence of envenoming with *Bothrops* snakes in Brazil (2012–2021). A) Map indicating the cumulative occurrence rates for the study period according to the state. B) Map indicating the cumulative occurrence rates for the study period according to region. C) Map showing the average incidence rates calculated by state (*envenoming/100,000 inhabitants). D) Map showing the average incidence rates calculated by region (*envenoming/100,000 inhabitants). The shapefiles for the municipalities, states and country, used for the creation of this map, were extracted from the Brazilian Institute of Geography and Statistics (https://www.ibge.gov.br/geociencias/todos-os-produtos-geociencias/15774-malhas.html).

In Brazil, between 2012 and 2021, 202,604 cases of envenoming caused by *Bothrops* snakes were recorded. During this period, the largest number of cases was geographically concentrated in the North region, which accounted for 79,145 (39%), followed by the Northeast and Southeast, both with approximately 21% of the registered occurrences, with 42,794 and 42,409, respectively. In the Midwest region (21,683 cases or 11%) and South (16,573 cases or 8%) the lowest occurrences were recorded (Table 1 and Fig 1).

**Table 1:** Annual occurrence of envenoming with *Bothrops* snakes among the five regions of Brazil (North, Northeast, South, Southeast and Midwest) during 2012–2021.

Region	FU	2012	2013	2014	2015	2016	2017	2018	2019	2020	2021	Total	(%)
**North**	RO	348	337	404	394	375	369	464	478	486	419	4074	2%
	AC	242	283	284	343	325	321	330	348	354	339	3169	2%
	AM	1063	1216	1165	1023	1099	1287	1467	1754	1776	1800	13650	7%
	RR	295	281	275	242	257	349	404	358	302	280	3043	2%
	PA	4187	4650	4580	4507	4394	4486	4498	5007	5046	4790	46145	23%
	AP	251	251	329	296	286	349	394	459	367	400	3382	2%
	TO	690	602	581	603	588	440	536	612	595	435	5682	3%
	Total	7076	7620	7618	7408	7324	7601	8093	9016	8926	8463	79145	**39%**
**Northeast**	MA	1075	1032	1113	1084	752	835	970	1346	1462	1581	11250	5,6%
	PI	109	72	74	76	88	96	101	136	158	198	1108	0,5%
	CE	378	285	255	376	451	463	457	577	528	503	4273	2,1%
	RN	156	71	102	158	223	247	231	358	391	316	2253	1,1%
	PB	184	105	108	213	222	210	207	281	340	265	2135	1,1%
	PE	215	145	128	238	291	219	248	286	294	377	2441	1,2%
	AL	82	104	79	100	106	101	93	105	83	114	967	0,5%
	SE	54	49	34	105	50	77	59	84	48	72	632	0,3%
	BA	1913	1881	1581	1750	1779	1831	1560	1690	1899	1851	17735	8,8%
	Total	4166	3744	3474	4100	3962	4079	3926	4863	5203	5277	42794	**21%**
**Southeast**	MG	2609	2491	1799	1805	1567	2066	2126	2138	2173	1814	20588	10%
	ES	777	790	577	596	500	586	743	595	6	1	5171	3%
	RJ	503	476	396	452	430	498	522	534	489	372	4672	2%
	SP	1216	1078	1200	1188	1038	1318	1245	1228	1238	1229	11978	6%
	Total	5105	4835	3972	4041	3535	4468	4636	4495	3906	3416	42409	**21%**
**South**	PR	528	494	545	543	571	545	443	468	416	391	4944	2%
	SC	623	552	577	581	541	544	501	494	534	482	5429	3%
	RS	707	652	659	720	689	705	565	588	483	432	6200	3%
	Total	1858	1698	1781	1844	1801	1794	1509	1550	1433	1305	16573	**8%**
**Midwest**	MS	445	359	383	400	388	430	384	390	288	259	3726	1,9%
	MT	953	920	952	1061	1010	988	848	1083	971	853	9639	4,9%
	GO	767	674	663	661	752	868	722	766	867	971	7711	3,8%
	DF	64	60	48	58	65	63	64	53	65	67	607	0,3%
	Total	2229	2013	2046	2180	2215	2349	2018	2292	2191	2150	21683	**11%**
**Total**		20434	19910	18891	19573	18837	20291	20182	22216	21659	20611	**202604**	**100%**

**Fig 2:**
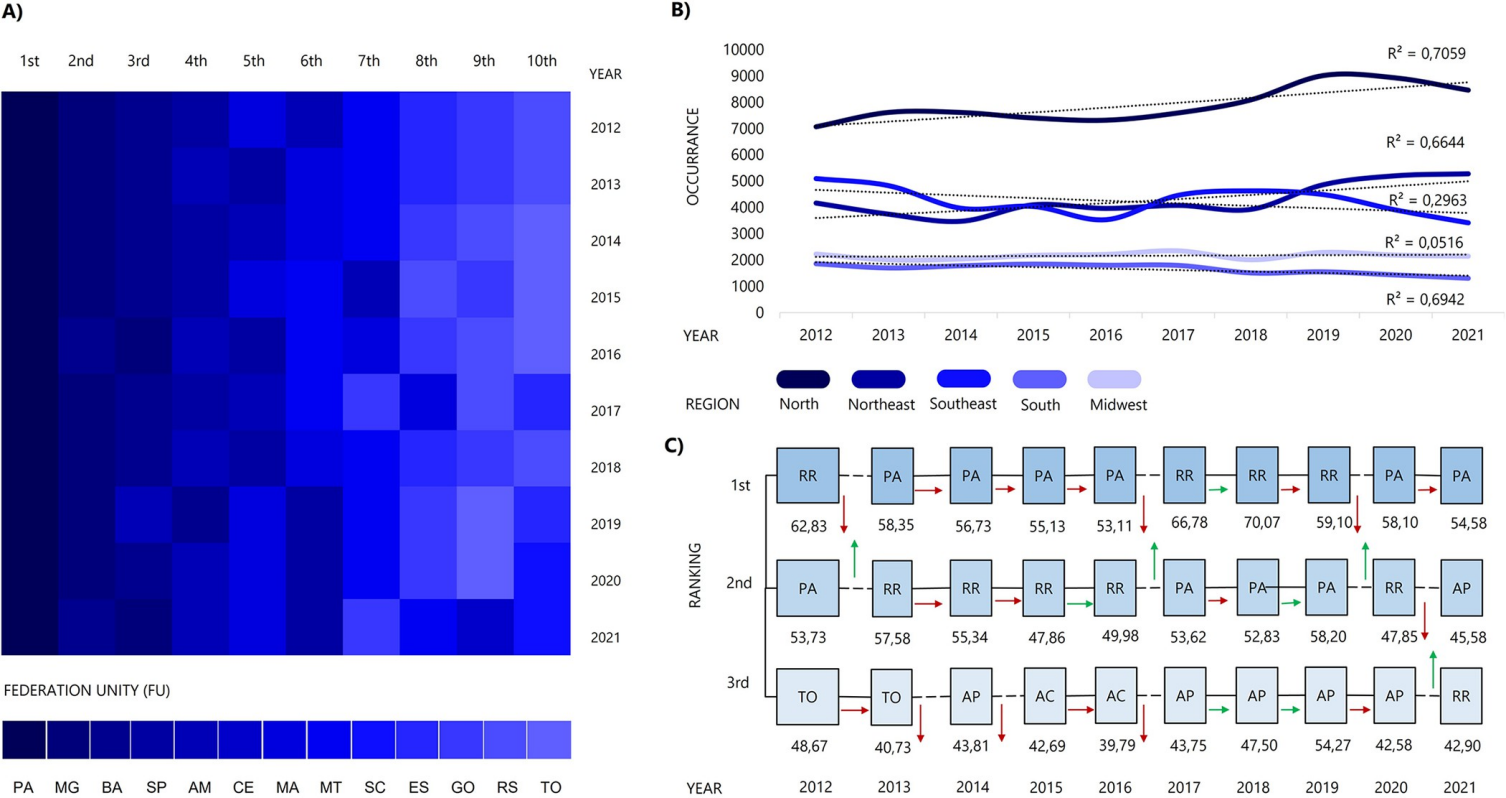
A) Ranking of states with the highest occurrences associated with *Bothrops* spp. between 2012 and 2021. B) Trends in the annual occurrence of *Bothrops* envenoming according to the region of the country. The R^2^ indicates how close the data are to the calculated linear regression line. C) Ranking of the 3 states with the highest incidence rates. The solid lines connecting the state names indicate that the state’s ranking position has been maintained from one year to the next, while the dashed lines indicate that the position has changed. Relative changes are shown according to annual incidence accompanied by green arrows indicating increase and red arrows indicating decrease.

To understand the epidemiological dynamics of reported envenoming in the five Brazilian regions, we proceeded with a ranking analysis based on the occurrence and incidence rates observed annually for each of the 27 federation units (FU) (Fig 2A and 2C). The states of Pará (PA), Minas Gerais (MG) and Bahia (BA) had the highest occurrences of envenoming with *Bothrops* snakes in the country in ten years (Fig 2A). Regarding the incidence, we identified noticeable differences between the North, Northeast, South, Southeast and Midwest, especially because the states Roraima (RO), Pará (PA) and Amapá (AP), three states in the north of Brazil, had an average incidence above 50 envenoming/100,000 inhabitants (Figs 1C and 2C).

In general, the mean incidence rates seen between 2012 and 2021 were similar in the Northeast, South and Southeast (6.09 to 7.51 envenoming/100,000 inhabitants, S2 = 0.65). Meanwhile, the incidence in the Midwest and, mainly, in the North reaches up to 40.94 envenoming/100,000 inhabitants (Fig 2B). Based on occurrence indicators, four Brazilian states occupy well-defined places in this ranking, Pará (1^st^), Minas Gerais (2^nd^), Bahia (3^rd^) and São Paulo (4^th^) (Fig 2A). Furthermore, according to the annual incidence of cases calculated by FU, we observe a scenario in which four states in the northern region (Amapá, Acre, Pará and Roraima) alternate among themselves in the first three positions (Fig 2C).

**Fig 3:**
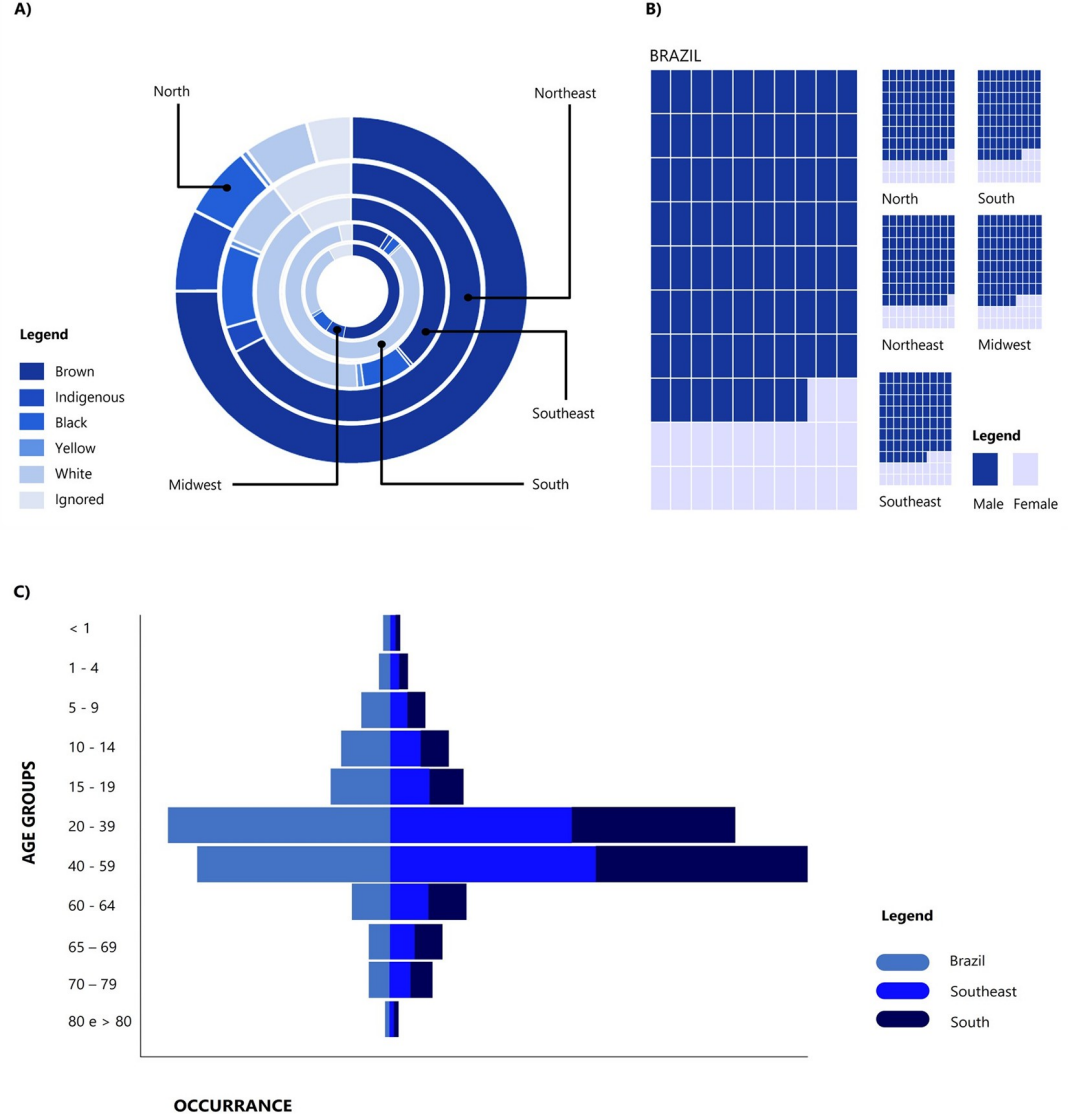
General characteristics of the population affected by *Bothrops* envenoming between 2012 and 2021 in Brazil. A) Ethnical characteristics of envenoming victims by region of the country. B) Population affected by envenoming according to gender (female or male). C) Age range of victims (groups: <1 year, between 1–4, 5–9, 10–14, 15–19, 20–39, 40–59, 60–64, 65–69, 70–79, 80 and >80 years).

In Brazil, the population affected by *Bothrops* spp. is composed of brown, black, white, yellow, indigenous individuals, and another portion whose color/race was not properly identified during medical care, and therefore is classified as ignored or blank. In the North, Northeast and Midwest of the country, 74.92%, 67.38% and 53.08% of the envenoming population are identified as brown, respectively. While in the South (83.70%) and Southeast (41.94%), those most affected by the disease self-identify as white. Furthermore, in all the regions, the group that suffers least from *Bothrops* spp. envenoming is yellow (the average for the 10 years among the five regions was 0.82%) (Fig 3A).

Regarding the sex of the envenoming patients, we identified a significant difference across the country (x^2^ = 61273, p < 0.0001), where 77.51% of the affected population is composed of men while only 22.49% are women, and, infrequently, records that did not identify the patient’s gender were found (0.0177%). Also, according to the region of the country, we found that in the North (x^2^ = 26493, p < 0.0001) and Northeast (x^2^ = 14003, p < 0.0001) approximately 79% of the patients were male and 21% to the female sex; in the South (x^2^ = 4876, p < 0.0001) and Southeast (x^2^ = 11951, p < 0.0001) 77% were male and 23% female, and in the Midwest (x^2^ = 5748, p < 0.0001) our results pointed to 76% of the victims being male and 24% female (Fig 3B).

According to the classification of the SINAN, patients are identified in age groups that comprise the blank/ignored groups (IGN), <1 year, between 1–4, 5–9, 10–14, 15–19, 20–39, 40–59, 60–64, 65–69, 70–79, 80 and >80 years. In Brazil, we found significant differences between age groups (H = 106.9, x^2^ = 495.5, df = 90, p < 0.0001), with more cases of envenoming by *Bothrops* spp. occurring in the groups aged 20 to 39 (34%) and 40 to 59 years (30%) (Fig 3C). According to the region of the country, in the North and Northeast the group aged 20 to 39 is the one that suffers most from envenoming (38% and 33%, respectively), while in the South and Southeast the group aged 40 to 59 years is the most affected by envenoming.

We also verified that between 2012 and 2021, 766 deaths resulting from *Bothrops* spp. were recorded by the SINAN in Brazil. The North and Northeast regions concentrate more than half of the cases in which the patient’s clinical condition evolved to death (n = 282, 37% and 214, 28%), while in the South only 7% of the cases that had death as an outcome (n = 55), and in the Southeast and Midwest 15% and 13% of envenoming, respectively, were fatal. Furthermore, the average mortality and lethality rates (CFR) in the period were 0.053 deaths/100,000 inhabitants and 0.37% for the entire country. These results varied according to the region and year throughout the studied period. However, comparatively, mortality was on average up to seven times higher in the North region than in the other regions, which presented similar results (Table 2).

**Table 2:** Access fatality rates (CFR)/100,000 inhabitants in % in Brazil according to region of the country.

Case Fatality Rate (CFR) %											
**Region**	**2012**	**2013**	**2014**	**2015**	**2016**	**2017**	**2018**	**2019**	**2020**	**2021**	**Total**
North	0,19	0,14	0,14	0,09	0,16	0,10	0,08	0,18	0,14	0,18	0,14
Northeast	0,04	0,02	0,02	0,03	0,04	0,03	0,04	0,04	0,03	0,04	0,03
Southeast	0,01	0,02	0,02	0,02	0,02	0,01	0,02	0,01	0,01	0,02	0,02
South	0,02	0,02	0,02	0,02	0,01	0,02	0,02	0,01	0,01	0,03	0,02
Midwest	0,06	0,06	0,06	0,05	0,05	0,08	0,06	0,06	0,06	0,06	0,06
**Total**	0,06	0,05	0,05	0,04	0,06	0,05	0,04	0,06	0,05	0,07	0,053

### 3.2 Clinical-epidemiological profile of accidents with *Bothrops* in Brazil

Most of the victims of *Bothrops* spp. were bitten on the feet (n = 49.3%), legs (n = 19.2%) and hands (n = 11.7%). According to the region of the country, there were no significant differences for these results, therefore during the studied period, the top 3 most affected body sites across all regions were the feet in first place, legs second and hands third (Table 3).

**Table 3:** Anatomical location of the bite in Brazil (2012–2021), according to region of the country.

Affected part of the body / Region	Head	Arm	Forearm	Hand	Finger	Stem	Thigh	Leg	Feet	Toe	Ignored	Total
North	868	1219	774	6398	2452	377	849	18164	42830	4632	582	79145
Northeast	475	757	504	5299	3164	184	225	5660	21863	3903	760	42794
Southeast	467	935	892	6907	4075	210	302	7256	17932	3047	386	42409
South	171	384	335	2542	1548	98	137	3275	6803	1076	204	16573
Midwest	209	394	306	2657	1356	97	169	4628	10363	1313	191	21683
Total	2190	3689	2811	23803	12595	966	1682	38983	99791	13971	2123	202604

Among the local manifestations and complications observed in patients, Table 3 indicates that pain ($\bar{x}$: 89%) and edema ($\bar{x}$: 77%) were the most frequent manifestations, while secondary infection ($\bar{x}$ 3%) represented the major cause of local complications in the clinic. Regarding systemic manifestations and complications, vagal (vomiting and diarrhea) ($\bar{x}$: 6%) and hemorrhagic manifestations ($\bar{x}$: 4%) were the most common among patients, as the main systemic complications were related to renal failure ($\bar{x}$: 0.8%) (Table 4).

**Table 4:** Symptomatology of envenoming by *Bothrops* snakes in Brazil (2012–2021), classified into local manifestations and complications and systemic manifestations and complications.

Symptomatology / Region	North		Northeast		Southeast		South		Midwest		Brazil
	Yes	(%)	Yes	(%)	Yes	(%)	Yes	(%)	Yes	(%)	Total
**Local Manifestations**											
Pain	72625	92%	35220	82%	38632	91%	14898	90%	19395	89%	89%
Edema	64195	81%	30311	71%	31865	75%	13236	80%	16483	76%	77%
Ecchymosis	9854	12%	4252	10%	7314	17%	3692	22%	3252	15%	15%
Necrosis	1175	1%	574	1%	613	1%	514	3%	361	2%	2%
Others	2644	3%	3337	8%	2676	6%	1603	10%	1603	7%	7%
**Local Complications**											
Secondary Infection	2978	3,8%	830	1,9%	937	2,2%	480	2,9%	761	4%	3%
Extensive necrosis	508	0,6%	178	0,4%	256	0,6%	195	1,2%	186	1%	1%
Compartment syndrome	587	0,7%	109	0,3%	223	0,5%	87	0,5%	207	1%	1%
Functional deficit	524	0,7%	141	0,3%	212	0,5%	143	0,9%	133	1%	1%
Amputation	50	0,1%	30	0,1%	25	0,1%	16	0,1%	18	0,1%	0,1%
**Systemic Manifestation**											
Paralytic	3194	4,0%	1789	4,2%	1050	2,5%	392	2,4%	729	3%	3%
Hemorrhagic	4277	5,4%	1976	4,6%	1553	3,7%	450	2,7%	675	3%	4%
Vagal	4916	6,2%	2266	5,3%	2302	5,4%	715	4,3%	1582	7%	6%
Hemolytic	1636	2,1%	837	2,0%	756	1,8%	382	2,3%	476	2%	2%
Renal	1215	1,5%	616	1,4%	479	1,1%	312	1,9%	463	2,1%	2%
**Systemic Complications**											
Renal insufficiency	342	0,4%	259	0,6%	312	0,7%	184	1,1%	266	1%	1%
Acute Pulmonary Edema	160	0,2%	88	0,2%	136	0,3%	59	0,4%	71	0,3%	0,3%
Sepsis	98	0,1%	31	0,1%	39	0,1%	18	0,1%	19	0,1%	0,1%
Shock	180	0,2%	80	0,2%	83	0,2%	46	0,3%	51	0,2%	0,2%

### 3.3 Temporal therapeutic itinerary in Brazil: average time elapsed between *Bothrops* snakebite envenoming and first medical care

**Fig 4:**
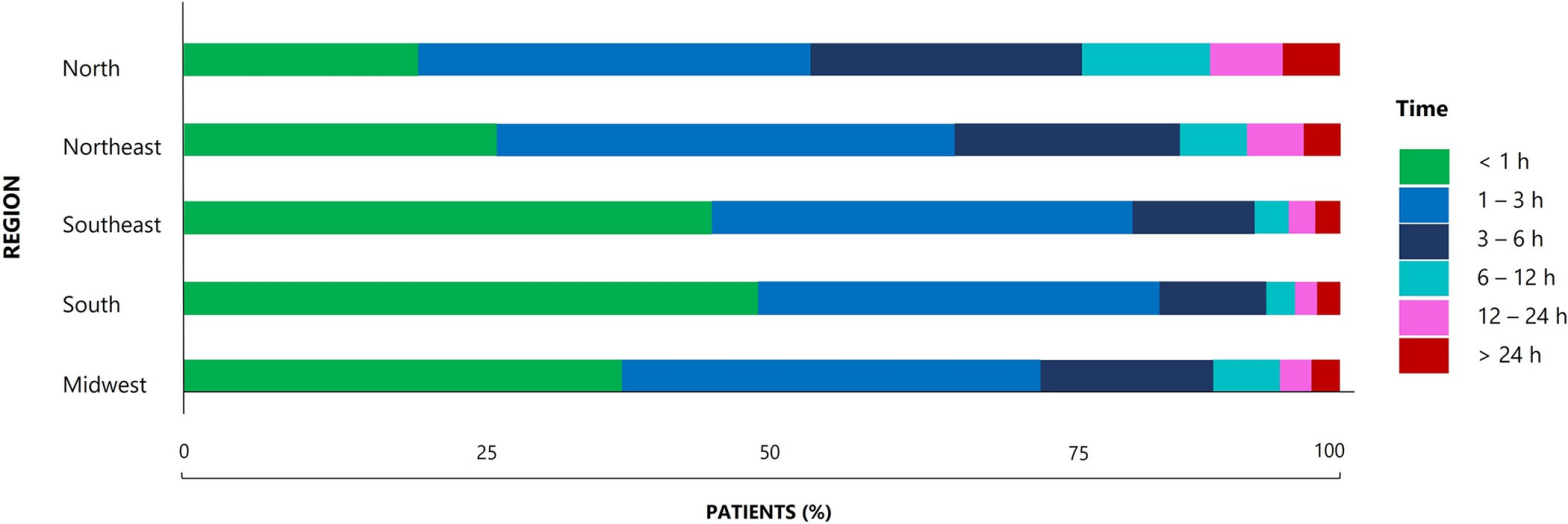
Temporal itineraries of patients bite by *Bothrops* spp. graphically expressed by region from the moment of the bite until hospitalization (2012–2021). The different colors of the fragments of each itinerary represent the time that the patients took from the moment of the bite until admission to the toxicological center that provided adequate Antivenom treatment.

The average duration from the accident with the snake to initial medical care was estimated to be around 3 hours for approximately 65% of snake envenoming cases attributed to the *Bothrops* genus. However, it is important to note that variations were observed across different regions of the country, as expressed in Fig 4, where the number of cases are depicted as a percentage. In the South and Southeast of the country, more than 40% of cases were seen before the first hour after envenoming, especially in the South region, where this percentage was 48%.

In contrast, in the North and Northeast, 19% and 26% of the population, respectively, arrive at the toxicological care center to receive care before the first hour after envenoming. In these regions, most patients can take up to 6 hours to receive adequate medical attention to treat the envenoming. Furthermore, most patients in the Midwest region are admitted within 3 hours after the accident with the snake occurred (Fig 4).

**Fig 5:**
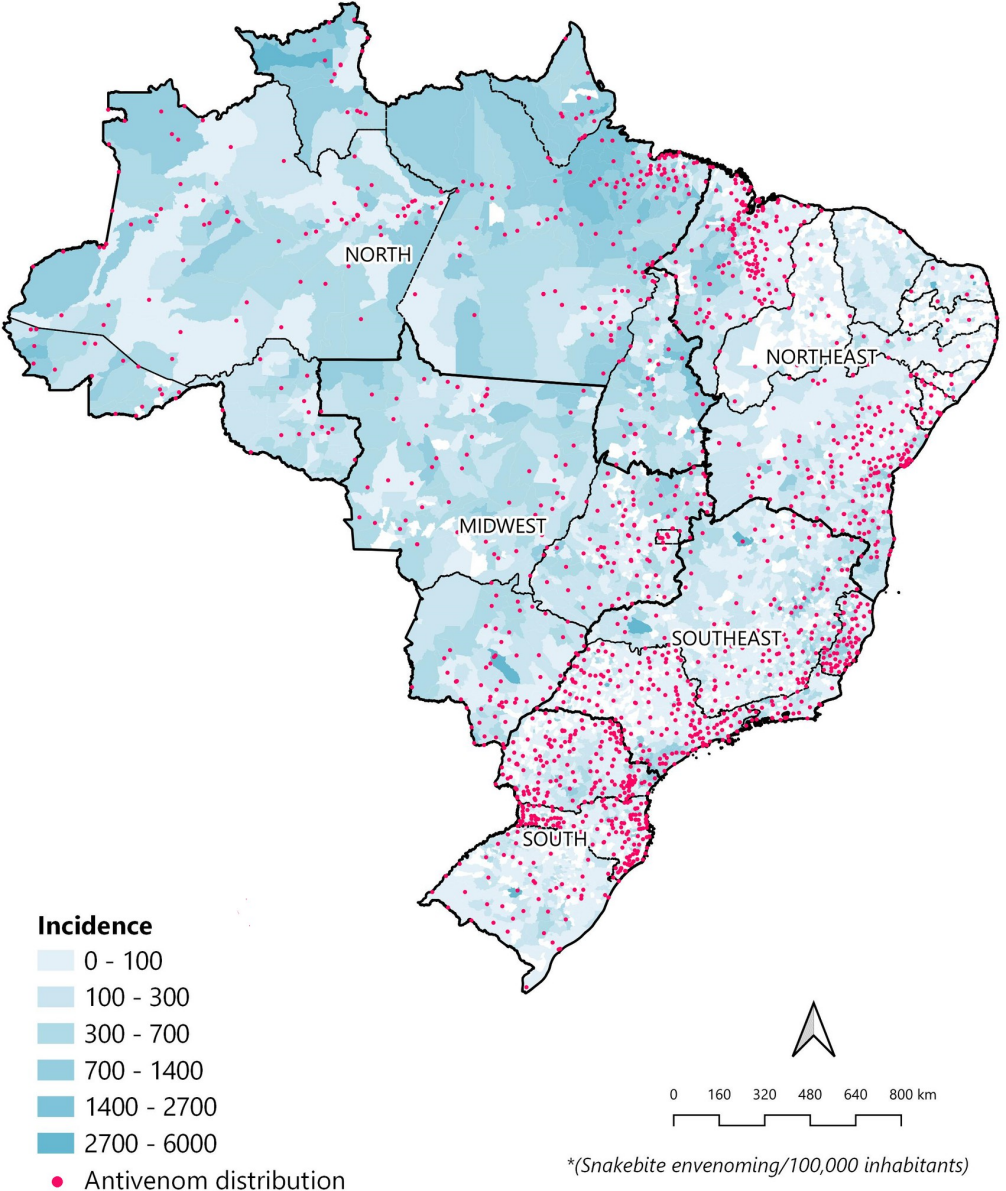
Spatial distribution of reference health centers for handling *Bothrops* snakebites in Brazil, by incidence of envenoming per municipalities and regions. The shapefiles for the municipalities, states and country, used for the creation of this map, were extracted from IBGE (https://www.ibge.gov.br/geociencias/todos-os-produtos-geociencias/15774-malhas.html). And the shapefile for the points representing the antivenom distribution were created by the authors using the addresses available at https://www.gov.br/saude/pt-br/assuntos/saude-de-a-a-z/a/animais-peconhentos/hospitais-de-referencia-para-atendimento.

To address and show key aspects of our work and broaden the possible real-world implication of the temporal itineraries of patient’s bites, we create a map that encompasses snakebites incidence by municipalities, along with punctuated distribution of Antivenom in all municipalities of Brazil. The results are shown in Fig 5 and indicates a disproportionate spatial distribution of health centers responsible for the distribution of the Antivenom in Brazil, with a concentration of many of these units in the South and Southeast, while in the North and Northeast regions, which concentrate most of the *Bothrops* envenoming, have a reduced amount these health centers capable of treating envenoming patients in a timely manner.

## 4. Discussion

Between 2012 and 2021 in Brazil, 202,604 cases of *Bothrops* snakebites were reported. This is equal to an average occurrence of 20,260 envenoming every year, aligning with what has already been seen in other studies of epidemiological surveys carried out in the country for other periods ($\bar{x}$: 20,000 envenoming/year) [13,60]. Most of the accidents were concentrated in the North (79,145 cases, $\bar{x}$: 7,914 envenoming/year), while a small number of envenoming were registered in the South (16,573 cases, $\bar{x}$: 1,657 envenoming/year), as has been shown by Schneider [61].

A study carried out by Chippaux [2], based on secondary data found in several Health information systems used by American countries, indicated that, in Brazil, between 2007 and 2012, the geographic distribution of the occurrence of envenoming with snakes showed a clear predominance in the North region, especially in the Amazon area, encompassing the states of Amazonas, Acre, Amapá, Rondônia, Pará and Roraima. However, the occurrence of envenoming according to snake group was not measured, which may be associated with the difficulty in handling a large amount of data for all snake groups that occur in a country such as Brazil [50,62].

Pará led the occurrence of envenoming in the North region during 2012 and 2021, concentrating almost 1/4 of all envenoming with *Bothrops* spp. These results are in line with what Magalhães et al. [63] demonstrated in an epidemiological study in the Amazon region, where between 2010 and 2015 the record cases in the SINAN indicated that most accidents with snakes occurred in the state of Pará (30,693 cases, 43.34%). Several explanations can be triggered to collaborate to this fact. One of them is the diversity in the ecological characteristics of Pará, such as climate conditions, vegetation and large presence of *Bothrops atrox* near inhabited areas, mainly in the rainy season of the year, when the flooding of rivers drives snakes to seek drier places [64], which increases the risk of envenoming [63,65].

At the same time, when we look at Minas Gerais, this state surprisingly takes the second place in the occurrence of envenoming. Possible reasons for the high rates of envenoming by *Bothrops* spp. in this state have not been previously addressed in the literature, however we can suggest some explanation for that. First, despite Minas Gerais is geographically situated in the Southeast of Brazil, the North of the state borders the Northeast of Brazil, and as well-known this region presents high temperatures throughout the year, which align with precipitation rates observed in summer and in the end of spring season, can contribute to the increase of *Bothrops* envenoming as has been seen in others subequatorial countries [66–68].

On the other hand, characteristically, the North of Minas Gerais aggregates risk factors and exposure to snakebites due to its phenomenon of peripheralization of cities and low Human Development Index (HDI), resulting in deficiency in sanitary practices and lack of knowledge of population practices of care and prevention [66,69]. In general, these characteristics allied with the main employment activities in the region such as Agriculture and Livestock may be key determinants for *Bothrops* envenoming [66,70]. However, despite we do not have information about the environmental, socioeconomic and occupational situation of the patients to confirm this inference, we encourage that other epidemiological studies in this state must be carried out.

The upward trend in the occurrence of envenoming with *Bothrops* spp. observed over the years, mainly in the North of the country, may be linked to other factors that go beyond the increase in the occurrence of envenoming. In 2014, Bochner and collaborators [71] theorized that the increase in the occurrence of snakebites in Brazil must also be linked to the improvements that have been implemented in The Brazilian Health Information System regarding the collection, storage and online distribution of epidemiological data and, at the same time, improving access to health services for the victims of envenoming. Furthermore, to the same extent, between 2020 and 2021, for two regions (North and Southeast), a downward trend in occurrence was observed. Perhaps this reduction in the number of cases is related to the non-use of health care centers during Covid-19 pandemic, which devasted unequally the population of Brazil [72].

However, we emphasize that the number of cases of envenoming caused by *Bothrops* spp. between 2020 and 2021 are still under review, and notification errors may have occurred. Nevertheless, by the date of writing this article (May 2023), the numbers of envenoming had not been updated in the SINAN remaining those reported here. A similar situation was observed in a recent study that analyzed envenoming caused by scorpions in Brazil. In it, Guerra-Duarte et al. found that, although the number of reported scorpion accidents was lower in 2020 than in 2019, the number of deaths seems to have skyrocketed, which could be an effect of complications from envenoming not properly treated, since people were avoiding going to health care centers [73].

In 2018, the state of Roraima had the highest average annual incidence seen in Brazil between 2012 and 2021 (70.07 envenoming/100,000 inhabitants). This number can be considered quite high even for the northern region, which throughout the period maintained an average annual incidence above 50 envenoming/100,000 inhabitants, with a slight downward trend over the years. Probably, the observed decline in the incidence of *Bothrops* spp. in the region resulted from population growth, since the occurrence of cases remained approximately constant throughout the period. The incidence of snakebites has been correlated in part by the biology, behavior and abundance of these animals in a region, and on the other hand by human activities in the field that can put entire populations at risk [1,13,74].

For comparison, the average annual incidence of snakebite envenoming in Sri Lanka, a South Asia country with one of the highest incidences linked to snakebite envenoming in the world, corresponds to 180 envenoming/100,000 inhabitants [75]. In Northern Brazil, several municipalities have presented incidence rates of over 100 envenoming per 100,000 inhabitants/year, mainly in the states of Amazonas, Roraima, Pará, Amapá and Tocantins [37].

In a study carried out by Alcântara et al. [37] in the North region, it was evidenced that in Roraima, the municipality of Alto Alegre holds the record for highest incidence rates of envenoming with snakes (358.3 envenoming per 100,000 inhabitants/year), followed by the municipalities of Anajá (338.9 per 100,000 inhabitants/year) and Afuá (303.7 per 100,000 inhabitants/year), in Pará. This reality may be directly linked to the population size of each of these municipalities, the climate conditions and the snake’s behavior, and the lack of use of personal protective equipment (PPE) in the daily activities, and not just because of the increase in the frequency of accidental encounters between snakes and humans.

However, these high incidence rates are restricted to a few municipalities, and therefore we can consider that *Bothrops* envenoming are unevenly distributed. According to World Health Organization [11], the incidence measure is rarely uniform in a country or region, since the distribution of snakes varies according to ecological, geographic and environmental parameters. Therefore, climate, human population density, transport networks, land use and habitat must be understood in a broader way [9,11].

The most affected racial groups were directly linked to the racial composition of the population in each of the regions, as well as in other parts of the world [9]. In the North of Brazil, approximately 70% of the population declares itself brown [51], in the Northeast this percentage is equal to 64% and in the Midwest 43%, while in the South and Southeast regions 74% and 59% of the population declare themselves white. Thus, in the North, Northeast and Midwest, the most affected by *Bothrops* spp. are brown, and in the South and Southeast white (r = 0.9320, P <0.0001). These findings contribute to other data already published regarding envenoming caused by other groups of snakes and venomous animals with medical importance in Brazil [60,76].

Furthermore, corroborating with previously published epidemiological studies, the population at risk in Brazil is formed mainly by males [2,13,60]. In addition, no significant differences were found for this variable between the regions studied, however, in the North and Northeast, approximately 79% of the patients envenomated by these snakes between 2012 and 2021 are men. This disproportion in the admission of male and female patients to toxicological care centers has been portrayed in the literature for decades as an occupational issue [9], since snake envenoming is closely linked to socioeconomic factors and professional occupation, the most affected being young people and agricultural workers, waste pickers, fishermen and working children [77–80].

In addition, according to the culture of certain countries, the female gender occupies a less prominent place in manual work, historically inherited by men, since women are limited to domestic tasks such as taking care of the house and the children, and therefore, they perform tasks with less risk of exposure to snakes. This information can be corroborated by a series of epidemiological studies such as those by Ceron [45,74], since both demonstrate, based on the SINAN database, that men are historically the most affected population by snakebite envenoming in Brazil.

Most of patients bitten by *Bothrops* spp. being between 20 and 59 years old must be linked to exposition to this group in daily activities [45], because this is the same age as a large portion of the active population that eventually can perform some function in the field, such as subsistence agriculture [45]. Age is another determining factor for snakebite envenoming, and in fact the involvement of the economically active population by envenoming caused by *Bothrops* spp. can be considered quite worrying, whether due to the temporary loss of labor in the field during the victim’s recovery period or even the inability of these workers to return to their jobs permanently due to chronic sequelae [81].

In this way, we can infer that envenoming caused by these snakes is much more a social, economic, and ecological problem than a medical one, as Fry [82] has articulated. Furthermore, unlike what was found by Chippaux [13], who considered all the genus of snakes that occur in Brazil, the specific incidence of envenoming caused by snakes did not increase with age up to 65 years and then decreased, nor did it show the same characteristics in all regions of Brazil. In fact, the occurrence of envenoming by the genus *Bothrops* in Brazil (2012–2021), showed an increasing trend from the first year of age up to 39 years, and then a clear decline, except for the South and Southeast, where the increase in occurrence was seen up to the 40–59 age group.

The 766 deaths notified between 2012 and 2021 and the geographic distribution of most of these notifications being concentrated in the North and Northeast highlight a problem that has been known for a long time, which unfolds mainly in the socioeconomic factors of these regions, and in the lack of attention given by government agencies to issues directly and indirectly related to the envenoming, such as government spending on health promotion, infrastructure, transportation and facilitating access to Antivenom [78].

These problems have had dramatic consequences for different locations, especially those that are more remote and distant from health care center [49]. An example of this is the current situation in the Amazon region, where long distances, low health coverage, the common use of ineffective or deleterious self-care practices and resistance to seeking medical assistance contribute to the mortality and lethality of envenoming with snakes [83], which illustrate our findings and could explain why the North region is precisely the one with the highest mortality rate, with an average equal to 0.14 deaths/100,000 inhabitants.

The anatomical site of the snakebite envenoming is an important variable that must be considered to tracing the clinical profile of the envenoming, since this is the “gateway” of the accident. In it is current global strategy for the prevention and control of snakebites, the World Health Organization has encouraged entire communities through education, especially those living in areas at risk of envenoming, to reduce the chances of snakebites occurrences with simple practices that can be adopted in everyday life, such as the use of shoes, boots and other objects capable of protecting the feet, ankles and legs [9].

There are many causes that culminate in snakebite envenoming. It is already well known that among the parts of the victims’ bodies, the extremities of the upper and lower limbs [80] such as feet, legs and hands are the most frequently affected [10]. According to our analyses, more often than not, regardless of the region of the country, most victims of *Bothrops* spp. presented the feet, legs and hands as the site of the sting, showing that envenoming must occur accidentally at inopportune times, and several studies have shown that this can be allied with the lack of the use of PPE in the rural areas and with the behavior of these snakes [65,84,85].

In Santa Cruz (Rio Grande do Norte, RN), a municipality considered a risk area for snakebites due to the already identified high annual incidence rate (up to 450 envenoming/100,000 inhabitants), accidents are almost always caused by *Bothrops erythromelas* [27] and most of the victims identified in an ethnozoological study carried out by Costa [85] claim to be residents of the rural area (77%) and perform some function in the field or in their own home at the time of the accident. In fact, the accidents reported by the interviewed patients occurred while working in the field or in the aviary, hunting in the mountains, caring for farm animals, while cleaning their home or surroundings [85].

In that same study, the most affected body region was the lower limbs, especially the feet. These results, in addition to being related to the activities performed by the patients, seem to be directly linked to the habits that these patients have in their daily lives, such as walking around unprotected or not paying attention while walking in regions where the risk of encountering a snake is high [84]. When questioned about the protection measures used to avoid accidents with snakes in the region, some patients in the municipality of Cruzeiro do Sul (Acre) reported some worrying measures ranging from (i) killing all snakes, (ii) carrying garlic and/or lemon in your pocket, (iii) carrying a weapon, (iv) faith in God, and (v) burning a bull’s horn. However, these strategies were not linked to measures that should provide physical protection to the victims’ bodies, such as (i) the use of boots, (ii) care and attention when walking, (iii) the use of long pants, (iv) not walking barefoot, (v) avoiding places considered dangerous such as dense forests, (vi) using other personal protective equipment [84].

*Bothrops* snakebites envenoming induces clinical manifestations such as pain, swelling, blister formation, myonecrosis, vascular damage, ischemia, necrosis, and coagulation abnormalities [86–91]. These clinical manifestations are attributed to the venom’s composition within this genus, primarily comprising Phospholipases A2 (PLA2s), Serine Proteases (SVSPs), Zinc-dependent Metalloproteases (SVMPs), L-Amino Oxidases (LAAO), Cysteine-Rich Secretory Protein (CRiSP), C-type Lectins (CTL), and Disintegrins (DIS) [19,20,92–94].

Although the high level of intraspecies variability among proteome of *Bothrops* venoms is recognized [18,92,95], the pattern of clinical manifestations, although of variable intensity, suggests the presence of a shared mechanism of tissue degradation, which may result in local complications such as: (a) compartment syndromes, (b) intense local and systemic tissue injury, (c) amputations [96–99], (d) shock, (e) acute pulmonary edema and (f) renal failure [88,100].

In Brazil, most of the envenoming patients between 2012 and 2021 reported pain at the site of the bite. In addition, edema was another local manifestation seen quite frequently in all five regions of Brazil, while ecchymosis and necrosis were present, but in smaller proportions. Following that, the most common systemic manifestations were linked to vomiting and diarrhea (vagal) and bleeding disorders, and systemic complications were rarely observed, in all regions the average of patients with systemic complications was less than 1%. These clinical manifestations are linked to the main mechanisms of action of *Bothrops* spp. venom, but also with the time between the envenoming and the administration of the Antivenom treatment, the use of traditional medicine and inadequate practices [10,65,82,97,98,101,102].

The time between envenoming and the first medical care is another important variable that is directly related to high rates of therapeutic success in the treatment of patients bites by *Bothrops* spp., that can be observed because the Antivenom therapies available can neutralize circulating toxins in human blood plasma [103]. Once that the success of treatment is closely tied to the promptness of medical care following snake envenoming. Therefore, the therapeutic itinerary taken by patients significantly influences their timely arrival at a toxicological care health center. Our findings indicate that, in the North region of Brazil, there is a higher prevalence of individuals affected by envenoming who arrive at the health care center after a span of 3 hours compared to other regions. These results further underscore the association between delayed medical attention and the region’s disproportionately elevated rates of mortality and lethality, which are up to 10 times higher compared to other regions of Brazil.

In a study that evaluated the therapeutic itinerary of 30 patients who lived in remote areas of the Amazon region [49], where access to Antivenoms is not guaranteed for these populations considered invisible [83], it was observed a great fragmentation in the itineraries of these patients, which was marked by several changes of means of transport along the route, in addition to the patients’ resistance to seek medical help. Some patients who lived more than 500 km away from the capital Manaus taking up to 96 hours to get to the toxicological care center, mainly due to the problems encountered during the journey, almost always done in motorboats, canoes, motorcycles, cars and planes [49].

Delay in medical care was reported by seventeen participants (57%) [49]. This profile was replicated, mainly, in the North and in the Northeast in 2012 and 2021. On the other hand, unlike that in the South and Southeast regions, throughout the period most patients arrived at the health care center even before completing the first hour after envenoming, which is directly related to better mobility conditions and accessibility to quality health services in these regions. While in the North and Northeast, two historically neglected regions of the country, a large percentage of patients took an average of up to 3 hours to arrive at the health care center that provided adequate Antivenom therapy to treat the envenoming.

## 5. Limitations

One significant concern pertains to the data collection process. All data obtained from SINAN is subject to review by the healthcare professionals responsible for providing and updating the epidemiological information in this system. The accuracy of this information is directly influenced by the process of identifying the biting snake, which depends on the memories of the patients and/or their relatives, or some record of the animal. Even when the animal is registered, there is often a lack of qualified agents to carry out an accurate identification of the snake at health centers, culminating in a possible sampling bias. Accurate identification of venomous snakes remains one of the biggest problems encountered worldwide, including in Brazil [104]. In addition, patients affected by *Bothrops* snakebite envenoming may decide not to go to the health centers to receive proper care, which masks the real epidemiological situation in the country and generates a large underreporting of cases. In this sense, we have provided an overview of the epidemiological situation *Bothrops* snakebite envenoming in Brazil, based on the data publicly available on SINAN. Although unfortunately our results may do not necessarily reflect the reality of the disease in the country, due to the limitations pointed out here, we believe that we have presented a careful approximation of this historical health problem, which disproportionately affects the population who lives in the shadows of neglect.

## 6. Concluding remarks

In Brazil, *Bothrops* snakebite envenoming is, in particular, an important cause of morbidity and mortality. Between 2012 and 2021, an extensive 202,604 instances of *Bothrops* snakebites were reported in SINAN. Our study findings demonstrated that the prevalence and incidence of envenoming were notably concentrated in the North region, followed by the Northeast and Southeast regions. The demographic characteristics of the population affected by *Bothrops* envenoming exhibited considerable heterogeneity based on the country’s regions. However, these outcomes consistently aligned with demographic data provided by IBGE. Notably, the North, Northeast, and Midwest regions reported higher proportions of patients self-identifying as brown, while in the South and Southeast regions, the majority identified as white. Moreover, a predominant male patient demographic was observed nationwide. Throughout the study period, there were 766 documented deaths attributed to complications arising from *Bothrops* spp. envenoming. More than half of these fatalities occurred in the North and Northeast regions, where delayed access to specialized care, particularly in rural areas, was prevalent due to the concentration of Antivenom in urban centers. This delay has been correlated with elevated mortality and lethality rates, as evidenced by other epidemiological studies focused on specific states within these regions. When comparing regions, the North emerged as the most region affected by *Bothrops* spp. envenoming, exhibiting annual mortality and lethality rates up to ten times greater than those observed in the South region.

Furthermore, our results revealed a consistent clinical profile among patients. This uniformity is attributed to the shared manifestations and clinical complications, primarily centered around pain, edema, and coagulation disorders induced by *Bothrops* venoms. Left untreated, these symptoms pose a significant risk of progressing into severe tissue damage and functional limb loss, often necessitating amputations. Our study underscored the vital role of a robustly functioning SINAN system and the pressing need for *Bothrops* envenoming to be mandatorily reported. This imperative action can pave the way for essential health indicators that aid in disease prevention and management. Moreover, the data prompts us to question the heightened intensity of *Bothrops* envenoming in remote rural areas, which often lack adequate healthcare facilities. Concurrently, there is a need to educate communities on appropriate actions to treat snakebite envenoming. This situation prompts us to advocate for an urgent call to action from authorities and health organizations to enhance therapeutic provisions, including the equitable distribution of Antivenoms in historically "invisible" areas such as the Amazon region.

## Supporting information

S1 Data Database organized by the authors from secondary data in the public domain by The Brazilian Notifiable Diseases Information System of the Brazilian Ministry of Health. (XLSX)

S2 Data List of Health care center that provides *Bothrops* Antivenom therapy on Brazil. (XLSX)
